# Comparison of Early Outcomes for Normothermic and Hypothermic Cardiopulmonary Bypass in Children Undergoing Congenital Heart Surgery

**DOI:** 10.3389/fped.2018.00219

**Published:** 2018-08-17

**Authors:** Antonio F. Corno, Claire Bostock, Simon D. Chiles, Joanna Wright, Maria-Teresa Jn Tala, Branko Mimic, Mirjana Cvetkovic

**Affiliations:** ^1^East Midlands Congenital Heart Centre, University Hospitals of Leicester, Leicester, United Kingdom; ^2^Cardiovascular Research Center, University of Leicester, Leicester, United Kingdom

**Keywords:** cardiac surgery, normothermia, hypothermia, cardiopulmonary bypass, pediatric intensive care unit

## Abstract

**Objective:** Comparison of early outcomes of normothermic cardiopulmonary bypass (N-CPB, ≥35°C) with hypothermic cardiopulmonary bypass (H-CPB, 28–34°C) for congenital heart defects.

**Methods:** Data from 99 patients <2 years operated with N-CPB (*n* = 48) or H-CPB (*n* = 51) were retrospectively reviewed: aortic X-clamping and CPB duration, vasoactive inotropic score (VIS), arterial lactate, pH and base excess, urine output, extubation, PICU stay, transfusion requirements, chest drain losses, costs of transfusions, and costs of PICU stay.

**Results:** The two groups were homogeneous for diagnosis, risk factors, surgery and demographic variables: N-CPB age 7.7 ± 6.1 months, weight 6.2 ± 2.4 kg, and H-CPB age 6.6 ± 6.5 months, weight 6.1 ± 2.4 kg.

There were no hospital deaths in either group. VIS in N-CPB was lower than H-CPB on PICU arrival (9.7 ± 5.9 vs. 13.4 ± 7.9, *P* < 0.005), after 4 h (7.0 ± 5.2 vs. 11.1 ± 7.3, *P* < 0.001) and 24 h (2.8 ± 3.6 vs. 5.6 ± 5.6, *P* < 0.003); arterial pH was better at PICU arrival (7.33 ± 0.09 vs. 7.30 ± 0.09, *P* = 0.046) after 4 h (7.35 ± 0.07 vs. 7.32 ± 0.07, *P* = 0.022) and after 24 h (7.37 ± 0.05 vs. 7.35 ± 0.05, *P* = 0.01). Extubation was earlier in N-CPB than in H-CPB (22 ± 27 vs. 48 ± 57 h, *P* = 0.003) as PICU discharge (61 ± 46 h vs. 87 ± 69 h, *P* = 0.021). Transfusion requirements in operating room were lower in N-CPB vs. H-CPB for RBC, FFP, cryoprecipitate, and platelets, while during the first 24 h in PICU were lower only for cryoprecipitate and platelets.

Chest drain losses (mL/kg) on PICU arrival, after 4 and 24 h were lower with N-CPB vs. H-CPB (respectively 1.5 ± 1.4 vs. 2.5 ± 2.7, *P* = 0.013, 7.8 ± 6.0 vs. 10.9 ± 8.7, *P* = 0.025, and 23.0 ± 12.0 vs. 27.9 ± 15.2, *P* = 0.043). Tranexamic acid infusion was required in 7/48 (14.6%) patients with N-CPB vs. 18/51(= 35.3%) in H-CPB (*P* = 0.009). The average total costs/patient of blood and blood products (RBC, FFP, cryoprecipitate, platelets) were lower in N-CPB vs. H-CPB for both the first 24 h after surgery (£204 ± 169 vs. £306 ± 254, *P* = 0.011) as well as during the total duration of PICU stay (£239 ± 193 vs. £427 ± 337, *P* = 0.001). The average cost/patient/day of stay in PICU was lower in N-CPB than in H-CPB (£4,067 ± 3,067 vs. £5,800 ± 4,600, *P* = 0.021).

**Conclusions:** N-CPB may reduce inotropic and respiratory support, shorten PICU stay, and decrease peri-operative transfusion requirements, with subsequent costs reduction, compared to H-CPB. Future studies are needed to validate and support wider use of N-CPB.

**Figure:**
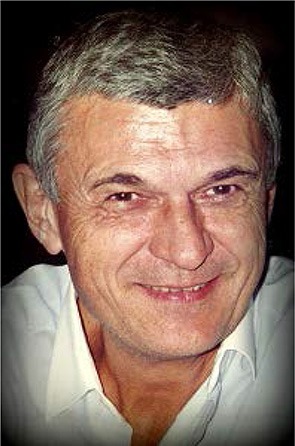
This paper is dedicated to the memory of Dr. Yves Durandy (1947–2016).

## Introduction

Surgery for congenital heart defects is generally performed with hypothermic cardiopulmonary bypass (CPB), reduced flow and hemodilution. This technique was historically first used to reduce the metabolic demand during CPB, and to increase the level of safety in the case of unforeseen complications (1).

In addition to the wide spread use of hypothermic CPB (H-CPB) with reduced flow and hemodilution, a large number of operations are still performed with deep hypothermia and circulatory arrest. This technique could be justified in small infants by the reduced duration of CPB, which is used only for cooling and rewarming, by the simplified venous cannulation, particularly in infants with anomalous venous connections, and by the bloodless field offering the surgeons adequate exposure (2).

Unfortunately H-CPB presents side effects, affecting all tissues and organs, with negative influence on clinical outcomes (1, 3–6). To minimize the negative effects of hypothermia, normothermic CPB has been progressively used for repair of congenital heart defects (4–6).

The aim of this retrospective study was to compare early clinical outcomes in patients less than 2 years of age with congenital heart defects who underwent cardiac surgery with normothermic (≥35°C) cardiopulmonary bypass (N-CPB) vs. hypothermic cardiopulmonary bypass (H-CPB).

## Methods

Clinical records of patients <2 years of age who underwent cardiac surgery for congenital heart defects with either N-CPB or H-CPB from January 2014 to December 2015 were retrospectively reviewed.

Exclusion criteria were:

- age >2 years
- premature patients (gestation age <38 weeks)
- operations performed without CPB
- operations performed with temperature lower than 28°C or with a period of circulatory arrest
- presence of aortic arch hypoplasia or interruption requiring aortic arch reconstruction.

Patients with operations performed at CPB temperatures lower than 28°C were excluded from this study to avoid introducing substantial flow reduction as a potentially important variable in the comparison.

In all patients included in this study the pump flow was maintained at 3.5 L/min/m^2^ B.S.A. in the group with N-CPB at a temperature always ≥35°C, while in the group with H-CPB the pump flow was reduced to 90% (= 3.15 L/min/m^2^ B.S.A.) at a temperature = 32°C and to 75% (= 2.63 L/min/m^2^ B.S.A.) at a temperature = 28°C.

The choice between the use of N-CPB and H-CPB was the decision of the surgeon in charge of the operation.

The following data were analyzed: demographic information, cardiac diagnosis, presence of previous median sternotomy, type of surgery, pre-operative risk evaluation accordingly with the STAT analysis, duration of aortic X-clamping and CPB, vasoactive inotropic score, arterial lactate, pH and base excess (B.E.) on PICU arrival, after 4 and 24 h, urine output (ml/kg/hr) after 4 and 24 h, time to extubation, duration of PICU stay, transfusion requirements of red blood cells (RBC), fresh frozen plasma (FFP), cryoprecipitate and platelets in operating room and during the first 24 h in PICU, chest drain losses on arrival to PICU and after 4 and 24 h, incidence of reoperations due to bleeding, the costs of blood and blood products used for transfusions in the first 24 h after surgery during PICU stay, as well as the cost of stay in PICU.

All data were expressed as mean ± Standard Deviation. As the small sample size did not allow for meaningful multivariable comparisons, a univariable comparison between the two groups was performed with Student's *T*-test, accepting as statistical significance *P* < 0.05.

The study received approval by the Institutional Review Board and Ethical Committee.

## Results

A total of 99 patients were identified, 48 with mean age 7.7 ± 6.1 months, mean weight 6.2 ± 2.4 kg, operated on with N-CPB, and 51 with mean age 6.6 ± 6.5 months, mean weight 6.1 ± 2.4 kg operated on with H-CPB.

The two groups, N-CPB and H-CPB, were homogeneous (no statistical difference) regarding the demographic variables of age (*P* = 0.20) and body weight (*P* = 0.36).

In the N-CPB group 2/48 (= 4.2%) of patients had a previous median sternotomy, one before bidirectional Glenn and the other before implantation of right ventricle to pulmonary artery conduit, and in the H-CPB group 5/51 (9.8%), three before repair of ventricular septal defect and two before bidirectional Glenn, but the difference did not reach statistical significance (*P* = 0.37).

Table 1 shows the distribution of cardiac diagnosis and type of surgical procedure between the two groups.

**Table 1:** Distribution of surgical procedures between the two groups.

**Surgical procedure**	**N-CPB**	**H-CPB**
Repair of ventricular septal defect	17	17
Repair of tetralogy of Fallot	9	11
Arterial switch for transposition of the great arteries	5	5
Repair of complete atrio-ventricular septal defect	2	5
Central shunt	4	1
Superior cavo-pulmonary connection	2	2
Repair of atrial septal defect	3	1
Repair of truncus arteriosus	1	3
Repair of total anomalous pulmonary venous connection	1	2
Right ventricular outflow tract reconstruction	2	0
Repair of cor triatriatum	1	2
Resection of sub-aortic fibromuscular ridge	0	1
Implantation of right ventricle to pulmonary artery conduit	1	0
Repair of mitral valve	0	1
Total	48	51

Pre-operative risk evaluation accordingly with the STAT analysis showed no difference between the two groups, with mean value 2.0 ± 1.2 in N-CPB and mean value 2.0 ± 1.1 in H-CPB (*P* = 0.46).

There was a statistically significant difference between N-CPB and H-CPB regarding the duration of aortic X-clamping, shorter in N-CPB vs. H-CPB (mean 58 ± 37′, vs. mean 76 ± 37′, *P* = 0.01) as well a shorter duration of CPB in N-CPB vs. H-CPB (mean 94 ± 41′, vs. mean 116 ± 41′, *P* = 0.005).

There were no hospital deaths in either group.

Table 2 shows the results of the early clinical outcomes for vasoactive inotropic score, arterial pH, lactate, duration of mechanical ventilation (time to tracheal extubation) and stay in PICU.

**Table 2:** Early clinical outcomes.

	**N-CPB**	**H-CPB**	***P* value**
**VASOACTIVE INOTROPIC SCORE**			
PICU arrival	9.7 ± 5.9	13.4 ± 7.9	<0.005
After 4 h	7.0 ± 5.2	11.1 ± 7.3	<0.001
After 24 h	2.8 ± 3.6	5.2 ± 4.9	<0.005
**ARTERIAL pH**			
PICU arrival	7.33 ± 0.09	7.30 ± 0.09	0.046
After 4 h	7.35 ± 0.07	7.32 ± 0.07	0.022
After 24 h	7.37 ± 0.05	7.35 ± 0.05	0.01
**LACTATE**			
PICU arrival	1.9 ± 1.1	2.9 ± 2.7	0.01
After 4 h	1.7 ± 0.6	2.4 ± 2.2	0.03
After 24 h	1.1 ± 0.5	1.6 ± 1.5	0.048
Mechanical ventilation (hours)	22 ± 27	48 ± 57	0.003
PICU stay (hours)	61 ± 46	87 ± 69	0.021

Although there was a trend in favor of N-CPB, no statistical difference was reached for arterial B.E. and urine output.

Table 3 shows the transfusion requirements for RBC, FFP, platelets, and cryoprecipitate, as well as the chest drains losses, in the operating room and during the first 24 h in PICU.

**Table 3:** Transfusion requirements and chest drains losses.

	**N-CPB**	**H-CPB**	***P* value**
**IN OPERATING ROOM (mL/kg)**			
RBC	8.6 ± 7.0	12.2 ± 7.0	0.007
FFP	3.4 ± 4.8	5.7 ± 6.8	0.034
Cryoprecipitate	1.3 ± 4.1	3.3 ± 5.9	0.031
Platelets	2.7 ± 4.9	5.1 ± 6.4	0.018
**FIRST 24 h IN PICU (mL/kg)**			
RBC	6.4 ± 9.5	7.0 ± 9.9	0.371 (NS)
FFP	4.6 ± 6.9	5.7 ± 6.8	0.202 (NS)
Cryoprecipitate	1.3 ± 3.2	2.7 ± 4.7	0.046
Platelets	1.9 ± 3.6	3.6 ± 4.9	0.029
**CHEST DRAINS LOSSES (mL/kg)**			
PICU arrival	1.5 ± 1.4	2.5 ± 2.7	0.013
After 4 h	7.8 ± 6.0	10.9 ± 8.7	0.025
After 24 h	23.0 ± 12.0	27.9 ± 15.2	0.043

There was no obvious correlation between chest drains losses and presence of previous median sternotomy: 1/2 patients with previous median sternotomy in N-CPB group and 2/5 in H-CPB group did not require any intra-operative or post-operative transfusion.

No statistical difference was reached in the incidence of reoperation due to chest bleeding, which occurred in 0/48 (= 0%) patients in N-CPB vs. 2//51 (= 3.9%) in H-CPB (*P* = 0.08).

In the group with N-CPB 3/48 (= 6.3%) of the patients required no transfusion vs. 2/51 (= 3.9%) in the group with H-CPB, but the difference failed to reach statistical significance (*P* = 0.30).

Tranexamic acid infusion was required in 7/48 (14.6%) of the patients with N-CPB vs. 18/51 (= 35.3%) in the group with H-CPB (*P* = 0.009).

The total costs per patient of blood and blood products (RBC, FFP, cryoprecipitate, platelets) were lower in N-CPB vs. H-CPB for both the first 24 h after surgery (mean £204 ± 169 vs. mean £306 ± 254, *P* = 0.011) as well as during the total duration of PICU stay (mean £239 ± 193 vs. mean £427 ± 337, *P* = 0.001).

The average cost/patient/day of stay in PICU was lower in N-CPB than in H-CPB (mean £4,067 ± 3,067 vs. £5,800 ± 4,600, *P* = 0.021).

## Discussion

### Hypothermia, flow reduction and hemodilution

Despite positive outcomes reported with H-CPB in surgical repair of complex congenital heart defects (7), several experimental and clinical studies over the years have demonstrated serious negative effects of hypothermia with flow reduction and hemodilution at both the cellular and tissue level (1, 3–6, 8–32).

Hypothermia, flow reduction and hemodilution have been demonstrated to decrease ATP levels, glycogen levels, intracellular pH, efficiency of membrane-based ion pumps, mitochondrial function, and intracellular enzyme function, as well as increased anaerobic metabolism, lactate production, cell swelling, and increased Calcium influx (1, 3–6, 8–32).

These changes in parenchymal cells, endothelial cells, and inflammatory cells are responsible for the inflammatory response and the ischemia/reperfusion injury, with the observed clinical consequences of low cardiac output syndrome requiring inotropic support, pulmonary dysfunction requiring respiratory support, metabolic derangement with acidosis and renal failure, coagulation derangement with excessive chest bleeding, and neurologic complications and neurodevelopmental impairment (1, 3–6, 11, 14–19, 21).

### Introduction of normothermia

The reported negative consequences of H-CPB have motivated the search for an alternative modality for perfusion in the pediatric population.

The first substantial change introduced in clinical practice was to reduce the degree of hemodilution, with a higher hematocrit on CPB (28, 31).

However the most important modification to conventional CPB with hypothermia and hemodilution was the introduction of normothermic high flow CPB.

This technique of perfusion was first used in Paris, France, by Lecompte and Durandy (33), who later reported the use of their technique in a very large number of pediatric cardiac patients (34, 35). Visits to their hospital, with direct exposure to their technique, persuaded other surgeons to introduce this method in their own clinical practice, resulting in an increase in the number of units using normothermic high flow CPB with reduced hemodilution (1, 4–6, 36–42).

The basic principles of N-CPB are maintaining CPB flow at 3.5 L/min/m^2^ B.S.A., nasopharynx and rectal temperature between 35.0 and 36.5°C, and hematocrit ≥30%. These conditions are closer to normal physiology, where the systemic flow is 3.0–5.5 L/m^2^ B.S.A./min, the temperature 37°C, and the hematocrit 45% (6).

The CPB flow used in conventional H-CBP is generally reported at 2.0–2.4 L/m^2^ BSA/min or 100–120 mL/min/kg of body weight, and frequently is further reduced during the central part of the operation when requested by the surgeon to facilitate the surgical exposure, or even to circulatory arrest with deep hypothermia.

In this study only patients with operations performed at a temperature ≥28°C were included in the H-CPB group, to avoid introducing a substantial flow reduction as this is potentially a confounding variable in the comparison.

The total duration of CPB is shorter with N-CPB than with H-CPB because the period of cooling at the beginning of CPB and the rewarming at the end of CPB, required for H-CPB, is avoided in N-CPB. This alone can contribute to reducing the negative effects of CPB.

We have also observed a reduction in the duration of aortic X-clamping in N-CPB. This is probably due to the fact that aortic X-clamping was limited to the closure of intra-cardiac defects and the right heart reconstruction is performed with beating heart, while in the H-CPB group the entire procedure was performed with the aortic X-clamp on, knowing that the heart reperfusion could have been accomplished during the required rewarming period.

### Potential concerns

The three potential concerns of using N-CPB are the reduced margin of safety against potential incidents during CPB, inadequate surgical exposure and adequate neurological protection.

As demonstrated in this study, as well as reported by other hospitals, the clinical advantages provided in the immediate post-operative period by N-CPB in comparison with H-CPB outweigh all potential risks of incidents (1, 5, 6, 33–38).

Adequate surgical exposure can be achieved with appropriate venous cannulation and left heart venting, even in small cyanotic neonates with large collateral circulation and in the presence of anomalous venous connections (1, 5, 6, 33–38).

With regard to the neurological complications reported by the H-CPB, the N-CPB was associated with contradictory neurologic outcomes, varying from the safety in relationship to the neurodevelopmental status (42) to the observation with magnetic resonance imaging suggesting that normothermic perfusion is associated with few new lesions in comparison with the pre-operative investigations (43).

### Limits of this study

The limits of this study are the following:

- retrospective
- non-randomized
- single center
- relatively small sample size
- small sample size did not allow for meaningful multivariable comparisons.

One of our aims is to share our experience and stimulate prospective multi-center randomized controlled trials comparing the outcomes of N-CPB with H-CPB.

Our retrospective study was motivated by the literature report of two studies comparing N-CPB with H-CPB without evidence of substantial differences (44, 45), while a very large systematic review showed that N-CPB is at least as safe as the conventional H-CPB (46). To the best of our knowledge we are not aware of any study comparing the early clinical outcomes and the blood requirements in N-CPB with H-CPB in patients younger than 2 years operated on for congenital heart defects.

## Conclusion

This experience shows that N-CPB is safe and reproducible, reduces inotropic and respiratory support, shortens the duration of PICU stay, and decreases the peri-operative transfusion requirements, with subsequent costs reduction, compared with H-CPB.

Wider use of N-CPB in pediatric cardiac surgery should be taken into consideration, and prospective multi-center randomized controlled trials comparing the outcomes with H-CPB in a risk adjusted population of patients with complex congenital heart defects are required to support these results.

## Author contributions

AC: design of the study, analysis of the results and preparation of the manuscript; CB, SC, and JW: data collection and revision; M-TT: study design, revision of the manuscript; BM and MC: revision of the manuscript.

### Conflict of interest statement

The authors declare that the research was conducted in the absence of any commercial or financial relationships that could be construed as a potential conflict of interest.
